# Phyllosphere bacterial and fungal communities vary with host species identity, plant traits and seasonality in a subtropical forest

**DOI:** 10.1186/s40793-022-00423-3

**Published:** 2022-06-09

**Authors:** Mengjiao Li, Lan Hong, Wanhui Ye, Zhangming Wang, Hao Shen

**Affiliations:** 1 Guangdong Provincial Key Laboratory of Applied Botany, South China Botanical Garden/Center of Plant Ecology, Core Botanical Gardens, Chinese Academy of Sciences, Guangzhou, 510650 China; 2 College of Horticulture and Landscape Architecture, Zhongkai University of Agriculture and Engineering, Guangzhou, 510225 China; 3 Southern Marine Science and Engineering Guangdong Laboratory (Guangzhou), Guangzhou, 511458 China; 4 College of Resources and Environment, University of Chinese Academy of Sciences, Beijing, 100049 China

**Keywords:** Phyllosphere, Bacteria, Fungi, Community assembly, Plant trait, Host species identity, Microbiome, Subtropical forest

## Abstract

**Background:**

Phyllosphere microbes play important roles in host plant performance and fitness. Recent studies have suggested that tropical and temperate forests harbor diverse phyllosphere bacterial and fungal communities and their assembly is driven by host species identity and plant traits. However, no study has yet examined how seasonality (e.g. dry vs. wet seasons) influences phyllosphere microbial community assembly in natural forests. In addition, in subtropical forests characterized as the transitional zonal vegetation type from tropical to temperate forests, how tree phyllosphere microbial communities are assembled remains unknown. In this study, we quantified bacterial and fungal community structure and diversity on the leaves of 45 tree species with varying phylogenetic identities and importance values within a 20-ha lower subtropical evergreen broad-leaved forest plot in dry and wet seasons. We explored if and how the microbial community assembly varies with host species identity, plant traits and seasonality.

**Results:**

Phyllosphere microbial communities in the subtropical forest are more abundant and diverse than those in tropical and temperate forests, and the tree species share a “core microbiome” in either bacteria or fungi. Variations in phyllosphere bacterial and fungal community assembly are explained more by host species identity than by seasonality. There is a strong clustering of the phyllosphere microbial assemblage amongst trees by seasonality, and the seasonality effects are more pronounced on bacterial than fungal community assembly. Host traits have different effects on community compositions and diversities of both bacteria and fungi, and among them calcium concentration and importance value are the most powerful explaining variables for bacteria and fungi, respectively. There are significant evolutionary associations between host species and phyllosphere microbiome.

**Conclusions:**

Our results suggest that subtropical tree phyllosphere microbial communities vary with host species identity, plant traits and seasonality. Host species identity, compared to seasonality, has greater effects on phyllosphere microbial community assembly, and such effects differ between bacterial and fungal communities. These findings advance our understanding of the patterns and drivers of phyllosphere microbial community assembly in zonal forests at a global scale.

**Supplementary Information:**

The online version contains supplementary material available at 10.1186/s40793-022-00423-3.

## Background

The phyllosphere, the aerial surface of plants (mainly the leaf surface), is estimated to tally up to 6.4 × 10^8^ km^2^ in the world, and it forms a common and important habitat for large numbers of species of terrestrial microorganisms (e.g. 10^26^ bacterial cells) [1, 2]. The heterogeneous environment formed by the phyllosphere is conducive to the coexistence of diverse microorganisms, including prokaryotes (bacteria and, less frequently archaea) and eukaryotes (fungi, oomycetes, and, less frequently nematodes), as well as viruses [1–4] The phytosymbiosis of microbes is a typical symbiotic relationship [5, 6]. The phyllosphere microbes exhibit a wide range of metabolic and functional diversities, and they are important for both their host plants and ecosystems [7, 8]. Their effects on ecosystem functions were discovered early [4]. For example, Ruinen [9] found that the dinitrogen in the atmosphere fixed by phyllosphere bacteria of the genera *Beijerinckia* and *Azotobacter* could be directly absorbed by the leaves or mobilized by the rain and supplied to the roots, thereby affecting the growth of tropical plants. Afterwards, similar results were found for temperate trees [10]. Subsequent studies have shown that phyllosphere microbial communities play an essential role in nitrogen fixation, bioremediation, and biocontrol agents [8, 11–13]. Therefore, accessing the information of the “core microbiome” [14] is important to understand the roles of phyllosphere bacteria and fungi.

The old microbiological tenet “Everything is everywhere, but, the environment selects’’ developed by Baas Becking in 1934 is the first biogeographic postulate [15]. Microbial ‘‘species’’ may be everywhere due to their huge dispersal potentials, but their abundances are constrained by contemporary environmental context [16]. Thus, exploring the driving factors of the phyllosphere microbial community composition is of great significance to understanding their roles in forest communities and management of the function and health of forest trees [8, 17]. Recent studies have shown that phyllosphere microbes could influence the growth and function of the host [18], while host species identity and host traits could also affect the survival and community composition of bacteria [17, 19, 20]. Reports have also shown that the composition of phyllosphere microbial community is driven by host plant traits (e.g., leaf age, leaf nutrient concentrations, leaf dry matter content, and leaf mass per area) [18, 21], a number of other host (host genotype and species identity) [17, 22, 23] and environmental factors (season, ultraviolet light, relative humidity, soil and biotic factors such as pathogens, nematodes, or insects) [8, 11, 24, 25]. Among these, traits and seasonal factors are very important since the former drive the composition of microbial communities [17–19], and the latter result in changes in community composition and diversity of phyllosphere bacteria and fungi [26–29]. Although we have some knowledge of plant–microbe interactions on tree leaf surfaces [1, 30], most studies on such interactions have focused on endophytic fungi [31–33] and pathogens [34–36], which limits our knowledge of the complex dynamics at play for other organisms [17].

Most work on forest phyllosphere microbes has focused on bacteria and fungi in tropical forests [13, 18, 19, 37, 38] and bacteria in temperate forests [17, 39], but the phyllosphere microbial community assembly in subtropical forests is poorly understood. Moreover, although the drivers such as host status and traits have been quantified in tropical and temperate forests, what roles they might play in subtropical forests remains unexplored. Such a knowledge gap limits our understanding of global patterns of microbial structure and diversity and their responses to global environmental changes. Located in the transitional region of tropical and temperate areas, the lower subtropics are sensitive to climate change, such as uncertain precipitation patterns [40]. Lower subtropical evergreen broad-leaved forest is the climax vegetation type of subtropics and it is characterized as the transitional zonal vegetation type from tropical to temperate forests [41]. The lower subtropical evergreen broad-leaved forests are frequently subject to seasonal drought [41–43]; therefore, they have a high potential for studying the seasonal patterns of phyllosphere microbial community assembly.

In this study, within a 20-ha forest biodiversity monitoring plot that is characterized as a lower subtropical evergreen broad-leaved forest, we used high-throughput sequencing to quantify the seasonal patterns and driving factors of microbial community structure and diversity on the leaves of 45 tree species with different phylogenetic statuses and importance values. We hypothesized that (1) phyllosphere microbial communities in the subtropical forest share a “core microbiome”, (2) host species identity, plant traits and seasonality have different relative influences on phyllosphere bacterial and fungal community composition and diversity; and (3) microbial community assembly differs between wet and dry seasons.

## Materials and methods

### Study site

The study site is the Dinghushan (DHS) 20-ha lower subtropical evergreen broad-leaved forest dynamics plot in southern China [44]. This plot is within the Dinghushan National Natural Reserve (area: 1155 ha) in the suburb of Zhaoqing City, Guangdong Province, China (23° 09′ 21″–23° 11′ 30″ N, 112° 30′ 39″–112° 33′ 41″ E). The region is located at the Tropic of Cancer and is characterized by a typical south subtropical monsoon climate. The mean annual temperature is 20.9 °C, with monthly temperatures ranging from 12.6 °C (January) to 28.0 (July). The mean annual precipitation is 1927 mm, of which about 80% occurs from April to September (wet season; mean monthly precipitation > 200 mm). The mean relative humidity is 85%, but an apparent dry season is from October to March with mean monthly precipitation < 100 mm [42]. The altitude of the DHS plot is from 230 to 470 m. The soil types are mainly lateritic red soil and mountain yellow brown soil.

Within the DHS plot, all woody stems with diameter at breast height (DBH) ≥ 1 cm have been measured, mapped, tagged and identified to species every five years since its establishment in 2005 [45]. In the 2015 census, a total of 210 woody species belonging to 103 genera and 55 families with DBH ≥ 1 cm within the plot was recorded.

### Host plant species selection and sampling

According to the phylogenetic relationships [46, 47] and importance values of all the 210 tree species with diameter at breast height ≥ 1 cm within the DHS plot, we selected 45 representative host plant species in 33 families and 19 orders for our study (Additional file 1: Table S1). Based on a maximum likelihood analysis of sequence data of three DNA barcoding loci (*rbcL*, *trnH-psbA*, and *matK*) for 183 plant species within the DHS plot, a molecular phylogenetic tree was generated, rooted by *Ginkgo biloba*, *Podocarpus fleuryi* and *Pinus massoniana* as outgroups [46, 47]. Within the phylogenetic tree, a total of 45 species were selected according to their different evolutionary distances from the outgroups, combining with their importance values in the plot. Consequently, they include one gymnosperm species (*Pinus massoniana*) and 44 angiosperm species; the latter include one monocotyledon species (*Caryota ochlandra*) and 43 dicotyledon species. They vary from dominant species (e.g., *Castanopsis chinensis* and *Engelhardia roxburghiana*) to rare species (e.g., *Magnolia paenetalauma* and *Itea chinensis*). We sampled three to four individual trees per species, in July (wet season) and December (dry season), yielding a total of 358 samples. Each sample consisted of some branches with shade leaves cut from the subcanopy (2–10 m above ground) and 50–100 g fresh healthy leaves clipped from the branches. The leaves were put into sterile roll bags with surface-sterilized shears for collecting microbes, and the rest were taken back to the laboratory for measurements of wood density (WD) and leaf traits.

### Microbial community collection and sequencing

We collected microbes from the leaf surfaces following the protocols of Lambais, Crowley [37]. Briefly, under aseptic conditions, we added 100 ml potassium phosphate buffer (0.1 M, pH = 7.0, containing 1 µl Silwet L-77) into a 4 L plastic bag containing 50 g of fresh leaves per tree and sonicated. Then, we obtained microbial suspensions by centrifuging the buffer. The total DNA of the microbial suspensions was extracted using a PowerSoil DNA isolation kit (MoBio Laboratories Inc., USA) according to the manufacturer’s instructions and then stored at − 80 °C.

For bacteria, the V3 and V4 regions of the 16S rRNA genes were amplified by PCR using the metagenomic DNA extracted as template and specific bacterial primers of 338F and 806R (F: 5′-ACTCCTACGGGAGGCAGCA-3′, R: 5′- GGACTACHVGGGTWTCTAAT-3′). For fungi, the ITS1 regions were amplified by PCR using the metagenomic DNA extracted as template and specific fungal primers of ITS1F and ITS2 (F: 5′-CTTGGTCATTTAGAGGAAGTAA-3′ R: 5′-GCTGCGTTCTTCATCGATGC-3′). We used a two-stage PCR approach to prepare amplicon libraries for the high-throughput Illumina sequencing platform [48]. The first-round tailed PCR was conducted to amplify regions of interest, and overhang adapter sequence was used in the second-round PCR and paired-end sequencing on MiSeq. The second-round tailed PCR aimed to add indices adapter sequences, and dual-indexed sequences and adapter sequences were bound to flow cells. In the first round, thermal cycling consisted of the following conditions: 95 °C for 5 min (1 cycle), 95 °C for 30 s/50 °C for 30 s/72 °C for 40 s (25 cycles), and a final extension at 72 °C for 7 min. In the second round tailed PCR, thermal cycling consisted of the following conditions: 98 °C for 30 s (1 cycle), 98 °C for 10 s/65 °Cfor 30 s/72 °C for 40 s (10 cycles), and a final extension at 72 °C for 5 min. High-throughput pyrosequencing of the PCR products was performed on Illumina NovaSeq6000 at BioMarker Technologies Co., Ltd. (Beijing, China).

The raw image data files obtained by high-throughput sequencing were converted into the original sequence by Base Calling analysis, and the results were stored in the FASTQ file format. It contained the sequence information (Reads) and Reads quality information. Using FLASH software (version 1.2.11) [49], the Reads of samples were assembled by overlap, and the obtained assembling sequences were the Raw Tags. Using the Trimmomatic software [50] (version 0.3.3), the Raw Tags were filtered to obtain Clean Tags. We obtained the Effective Tags by using UCHIME software (version 8.1) [51] to identify and remove chimeric sequences. Then, we clustered the Tags to obtain operational taxonomic units (OTUs) at a 97% sequence similarity level by using UCLUST [52] in QIIME (version 1.8.0) [53] and classified OTUs based on the Silva (bacteria) and UNITE (fungi) taxonomic databases.

### Host plant traits

We selected 19 plant traits, mainly functional traits [54], to explore their effects on microbial community composition and diversity. They included average DBH, average tree height (height), importance value (IV), tree DBH relative growth rate (GR), tree mortality rate (MR), maximum CO_2_ assimilation rate per unit dry mass (A_mass_) and photosynthetic water use efficiency (WUE) from data previously collected from the DHS plot. Methods used to measure these traits are given in supplementary materials (Additional file 2: Table S2). Measurements of leaf morphological traits including specific leaf area (SLA) and leaf dry matter content (LDMC), leaf stoichiometric traits including concentrations of carbon (C), nitrogen (N), phosphorus (P), calcium (Ca), potassium (K) and silicon (Si), leaf defense traits including concentrations of phenolics (Phe), soluble tannins (Tan) and flavonoids (Fla), and sapwood density (WD), were conducted on the branches or leaves of the 358 samples.

### Statistical analyses

Data analyses and visualization were performed using the tidyverse [55], ape [56], ggplot2 [57], picante [58], vegan [59], gclus [60], phyloseq [61], reshape2 [62], dismo [63], paco [64] packages for R [65]. To reduce the errors caused by intraspecies differences, we combined the OTUs of all 3–4 samples per species per season and thus obtained a total of 90 combined samples (45 species × 2 seasons). Specaccum function was used to plot the species accumulation curve. To understand the effects of seasonality on microbial community composition and diversity, NMDS plot was generated by Bray–Curtis distance across the 90 combined samples. We partitioned the variance in phyllosphere microbial community structure explained by season and host species identity using variance partitioning and permutational multivariate analysis of variance (PERMANOVA) analysis on Bray–Curtis distances [66]. To quantify the influence of host taxonomic levels on bacterial community structure, we performed a nested PERMANOVA at the levels of order, family, genus and species. Based on the PERMANOVA results, detrended correspondence analysis (DCA) was applied to the data set, which revealed a gradient length of the first axis < 3 for either bacteria (2.38) or fungi (2.34), indicating a linear response[67]. Thus, redundancy analysis (RDA) was applied to analyze the influence of host plant traits on phyllosphere microbial community composition and core phyllosphere microbiome. We performed the multicollinearity tests before RDA to reduce redundant variables, and we found variance inflation factors of all the 19 plant traits were less than 10; therefore, all the 19 traits were used to conduct the RDA. Through Wilcoxon rank sum test, we obtained the differences in alpha diversity and species in different seasons. The relationships between biodiversity metrics and potential explanatory variables were further analyzed separately for bacteria and fungi using boosted regression trees (BRT) which is an ensemble method for fitting statistical models [63], for the whole data sets of 90 combined samples covering two seasons. For the biodiversity metrics, we selected Shannon diversity index and Pielou evenness index. We tested the overall evolutionary association between host plant species and phyllosphere bacteria/fungi and individual host-bacteria/fungi associations using the “host-parasite association test” [18, 68].

## Results

### Composition and diversity of phyllosphere microbial community and the core microbiome

We identified 22,236 bacterial OTUs in the 90 combined samples, with an average of 2432 ± 109 OTUs per sample (Additional file 4: Fig. S1a). Among these OTUs, the wet and dry seasons shared 10,918 OTUs, and the OTUs unique to the wet and dry seasons are 9196 and 2122, respectively (Additional file 5: Fig. S2a). For phyllosphere fungi, we identified 18,471 fungal OTUs, with an average of 2144 ± 706 OTUs per sample (Additional file 4: Fig. S1b). Among these OTUs, the wet and dry seasons share 9225, and the OTUs unique to the wet and dry seasons are 4807 and 4439, respectively (Additional file 5: Fig. S2b).

**Fig. 1:**
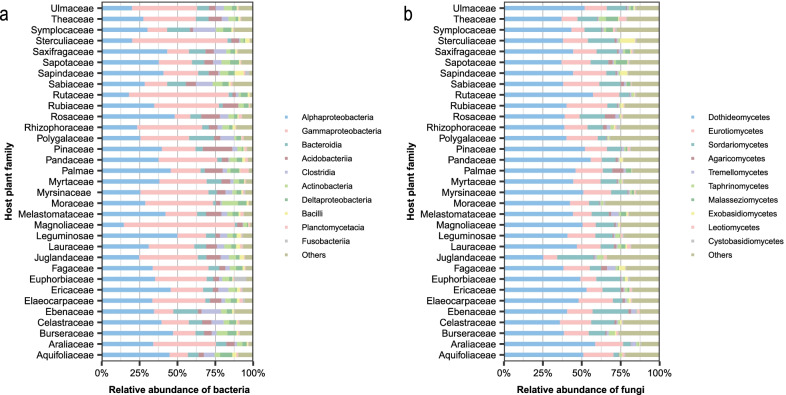
Taxonomic composition of phyllosphere microbial communities. Relative abundance of class-level taxonomic compositions of phyllosphere bacterial (**a**) and fungal (**b**) communities on different host plant families

We defined “phyllosphere core microbiome” consisting of OTUs that were present on 99% or more of all the species sampled. We observed that the phyllosphere core bacterial microbiome had 69 OTUs that accounted for 0.31% of the bacterial taxonomic diversity but more than 46.7% of sequences, in 4 phyla, 7 classes, 15 orders and 19 families. The five most dominant classes within it are *Alphaproteobacteria* (34.1%) [*Sphingomonadaceae* (11.8%), *Beijerinckiaceae* (14.2%), *Acetobacteraceae* (3.3%)], *Gammaproteobacteria* (31.3%) [*Enterobacteriaceae* (8.6%), *Pseudomonadaceae* (7.7%), *Moraxellaceae* (4.7%)], *Betaproteobacteria* (6.8%) [*Burkholderiaceae* (6.4%)], *Acidobacteriia* (6.2%) [*Acidobacteriaceae* (5.6%)], and *Clostridia* (4.6%) (Fig. 1a).

We found the phyllosphere core fungal microbiome consisted of 70 fungal OTUs representing 0.38% of the fungal taxonomic diversity but more than 70.28% of sequences (Fig. 1b) The most dominant fungi are Ascomycota (87.3%). The five dominant classes in the core microbiome are *Dothideomycetes* (44.32%), [*Aureobasidiaceae* (4.6%), *Dissoconiaceae* (13.88%), *Mycosphaerellaceae* (4.70%), *Teratosphaeriaceae* (1.38%), *Dothioraceae* (3.80%)], *Sordariomycetes* (10.8%) [*Sporocadaceae* (3.37%)], *Eurotiomycetes* (15.57%) [*Onygenales incertae sedis* (2.72%)], *Agaricomycetes* (1.48%), and *Tremellomycetes* (1.48%) (Fig. 1b).

### Microbial community composition and diversity in different seasons

**Fig. 2:**
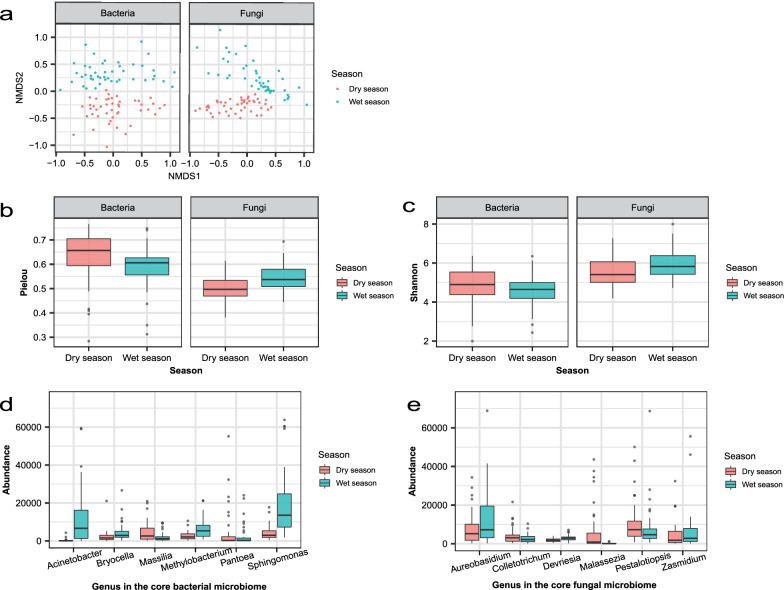
The community composition and diversity of phyllosphere microbes in dry and wet seasons. (**a**) Nonmetric multidimensional scaling (NMDS) ordination of variations in bacterial and fungal community structure (Bray–Curtis distance) in different seasons. Multi Response Permutation Procedure based on Bray–Curtis distance, bacteria: *p* = 0.001, fungi: *p* = 0.001. (**b**) Pielou evenness index and (**c**) Shannon diversity index of phyllosphere microbial communities in different seasons (Wilcoxon rank sum test). Only the bacterial Shannon diversity indices in dry and wet seasons are not significantly different. (**d**) Abundance in genus of the core bacterial microbiome between groups in different seasons (Wilcoxon rank sum test). (**e**) Abundance in genus of the core fungal microbiome between groups in different seasons (Wilcoxon rank sum test)

The community composition of either bacteria or fungi was different between wet and dry seasons (Fig. 2a). The NMDS results showed that there is a strong clustering of the phyllosphere assemblage amongst trees by seasonality. The results of Multi Response Permutation Procedure based on Bray–Curtis distance also showed that seasonality was a significant driver for microbial community composition of bacteria (*p* = 0.001) and fungi (*p* = 0.001).

Further analysis on community composition was performed using alpha diversity indices, including Shannon diversity index and Pielou evenness index. Since in the Shapiro–Wilk test, the two indices both did not show normal distributions, the Wilcoxon rank sum test was therefore chosen to analyze them. Our analyses indicate that Pielou index of bacterial community (Fig. 2b) is significantly lower in the wet season than in the dry season (*p* = 0.01). Pielou (*p* < 0.001, Fig. 2b) and Shannon (*p* < 0.05, Fig. 2c) indices of the fungal community are both significantly higher in the wet season than in the dry season.

For the same reason as in the analysis of the alpha diversity indices, we used Wilcoxon rank sum test to analyze the relative abundances of both bacterial and fungal core microbiomes. At the level of genus, for the bacteria (Fig. 2d), the abundances of *Acinetobacter*, *Bryocella*, *Methylobacterium* and *Sphingopyxis* are significantly higher in the wet season than in the dry season at *p* < 0.001, and the opposite is true for *Massilia* (*p* < 0.001), and *Pantoea* (*p* < 0.001). For the fungi (Fig. 2e), the richnesses of *Colletotrichum* (*p* < 0.05), *Malassezia* (*p* < 0.001), *Pestalotiopsis* (*p* < 0.05) are significantly higher in the dry season than in the wet season, and the opposite is true for *Devriesia* (*p* < 0.001), and *Aureobasidium* and *Zasmidium* (*p* < 0.05).

### Drivers of variation in phyllosphere microbial community composition and diversity

The results of PERMANOVA on Bray–Curtis distance showed that host species identity and season together explained a total of 43.19% (*p* < 0.001) of the variation in bacterial community structure, with host species identity, season, and their interaction accounting for 20.31% (*p* < 0.001), 6.49% (*p* < 0.001), and 16.39% (*p* < 0.001), respectively. The host taxonomic order, family, genus, and species, respectively, significantly (*p* < 0.001) explained 8.74%, 6.59%, 2.95%, and 2.03% of the variation accounted by host species identity. The same analysis showed that host species identity and season together explained a total of 50.38% (*p* < 0.001) of the variation in fungal community structure, with host species identity, season, and their interaction accounting for 36.79%, 1.97%, and 11.62% (*p* < 0.001), respectively. The host taxonomic order, family, genus, and species significantly (*P* < 0.001) explained 18.05%, 10.58%, 6.24%, and 1.92% of the variation accounted by host species identity, respectively.

**Fig. 3:**
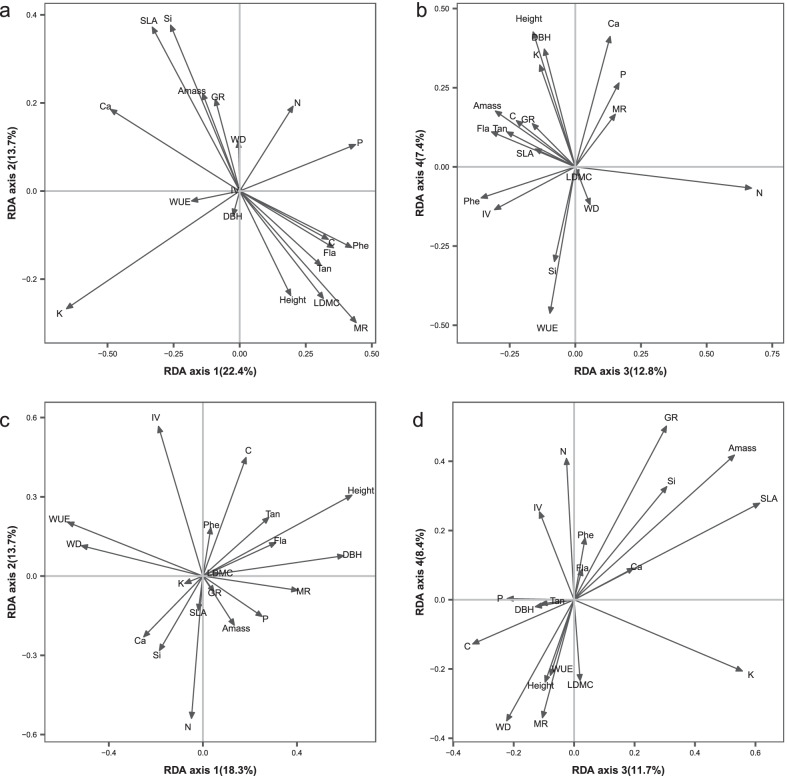
Redundancy analysis (RDA) on the relationships between microbial community structure and suites of correlated host traits across species. **a**, **b** RDA on the relationship between bacterial community structure and the 19 host traits. **a** Axis 1 versus 2; **b** Axis 3 versus 4. These four axes explained 57.3% of the variation in the data. **c**, **d** RDA on the relationship between fungal community structure and the 19 host traits. **c** Axis 1 versus 2; **d** Axis 3 versus 4. These four axes explained 52.1% of the variation in the data

In bacterial community structure, the results of RDA showed that the 19 host plant traits explained 8.44% (adjusted R-square; Permutation test, *p* = 0.001) of the variation, of which the first, second, third, and fourth RDA axes of the correlated traits explained 22.4%, 13.7%, 12.8%, and 7.4%, respectively (Fig. 3a, b). The first and second axes are associated mainly with growth and stoichiometric traits, including LDMC and SLA, and leaf nutrient concentrations (C, P, Ca, Si, K), and these traits are associated with the “leaf economics spectrum” of plant resource uptake strategies [69–72]. The third axis is mainly related to the defensive traits, including Phe concentrations, total soluble Tan, and total Fla. The fourth axis is mainly related to plant sizes, such as average height and DBH.

In fungal community structure, the results of RDA show that 19 host traits explained 15.63% (adjusted R-square; Permutation test, *p* = 0.001) of the variation, of which the first, second, third and fourth axes account for 18.3%, 13.7%, 11.7%, and 8.4%, respectively (Fig. 3c, d). The first axis relates to plant growth-mortality trade-off strategy, including average height and DBH, MR, WD, and WUE. The second axis is mainly associated with IV and stoichiometric traits including concentrations of C, N, and Ca that are associated with the “leaf economics spectrum”. The third axis is mainly related to SLA, K and A_mass_, and these are related to the growth and physiology of the plants. The fourth axis is mainly related to A_mass_, N concentration, WD, GR and MR.

**Fig. 4:**
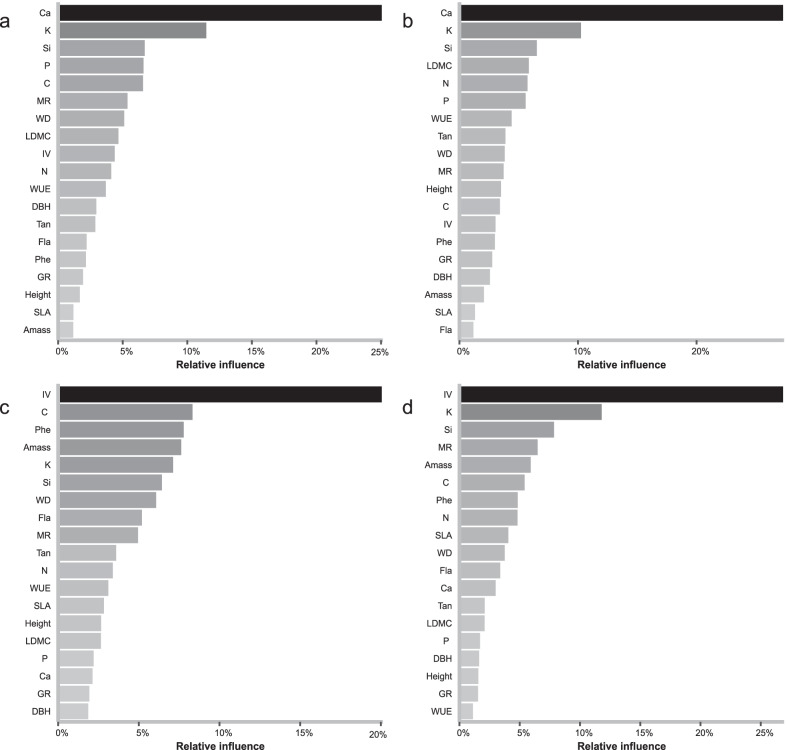
Relative influences of host traits on the Shannon index and Pielou index of microbial communities from Boosted Regression Trees. **a** Bacterial Shannon index. **b** Bacterial Pielou index. **c** Fungal Shannon index. **d** Fungal Pielou index. SLA, specific leaf area; LDMC, leaf dry matter content; A_mass_, maximum CO_2_ assimilation rate per unit dry mass; WUE, photosynthetic water use efficiency; C, leaf carbon concentration; N, leaf nitrogen concentration; P, leaf phosphorus concentration; Ca, leaf calcium concentration; K, leaf potassium concentration; Si, leaf silicon concentration; Phe, total leaf phenolics concentration; Tan, total leaf tannins concentration; Fla, total leaf flavonoids concentration; DBH, tree diameter at breast height, Height, average tree height; GR, DBH relative growth rate from the year 2005 to the year 2015; MR, tree mortality rate from the year 2005 to the year 2015; WD, sapwood density; IV, importance value

Based on the results of BRT, Ca concentration is the most powerful variable in explaining the variations in bacterial Shannon indices (relative influence of 25.1%) (Fig. 4a) and Pielou indices (27.4%) (Fig. 4b), and it is negatively correlated with them (*p* < 0.05) (Additional file 3: Table S3). For the Shannon indices, the other top four powerful explaining variables are K, Si, P, and C concentrations with relative influences of 11.5%, 6.70%, 6.61%, and 6.57%, respectively (Fig. 4a). For the Pielou indices, the other top four powerful explaining variables are K and Si concentrations, LDMC, and N concentration with relative influences being 10.3%, 6.53%, 5.85, and 5.74%, respectively (Fig. 4b).

For the drivers of the biodiversity of fungi, the results of BRT indicate that IV is the most important variable in explaining the variations in fungal Shannon indices (relative influence of 20.1%) (Fig. 4c) and Pielou indices (26.9%) (Fig. 4d), and it is negatively correlated with both (*p* < 0.05) (Additional file 3: Table S3). For the Shannon indices, the other top four important explaining variables are C concentration (8.33%), Phe concentration (7.78%), A_mass_ (7.63%) and K concentration (7.12%) (Fig. 4c). For the Pielou indices, the other top four important explaining variables are K concentration (11.8%), Si concentration (7.84%), MR (6.48%) and A_mass_ (5.91%) (Fig. 4d).

**Fig. 5:**
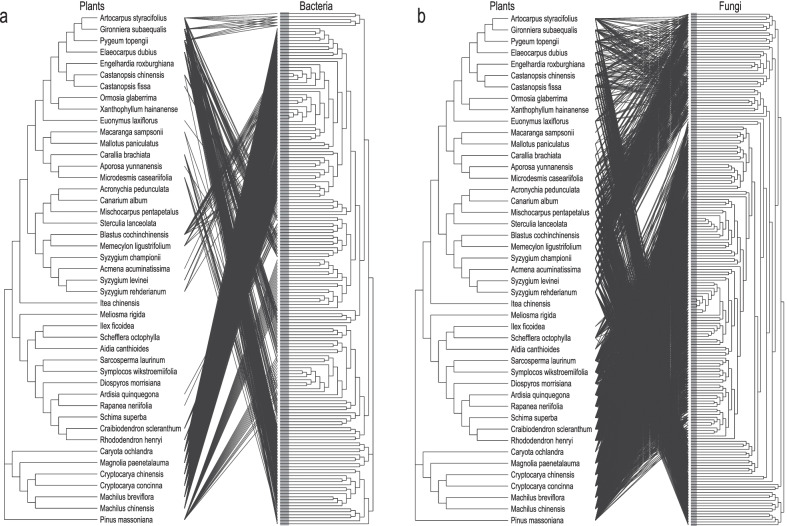
Cophylogeny of host plant species (left) and phyllosphere bacteria (**a**) and fungi (**b**) (right). Lines connecting tips on the phylogenies indicate significant plant-microbes associations according to a host-parasite coevolution test (*p* < 0.05)

Results of host-parasite association test show that there are significant (*p* < 0.05) overall evolutionary associations between host species and the OTUs of the bacteria (Fig. 5a) and fungi (Fig. 5b), and the numerous associations between host species and both bacterial (Fig. 5a) and fugal (Fig. 5b) clades.

## Discussion

### Composition and diversity of phyllosphere microbial community and the core microbiome

As the transitional region of tropical and temperate areas, the lower subtropics act as a biota exchange center and therefore contain both tropical and temperate species [44, 73]. This might be the reason that both total OTUs and mean OUTs per plant within the phyllosphere bacterial and fungal community are respectively greater than those in tropical and temperate forests [17, 18], and in tropical rainforests [19]. In temperate forests, the phyllosphere of *Quercus macrocarpa* in rural and urban environments was found to support less fungal OTUs than the subtropical forest in this study [74]. Thus, the high diversities we observed for both bacteria and fungi together suggest that the phyllosphere of subtropical forests supports a more diverse microbial community than those of tropical and temperate forests do.

In our study, the tree species in the lower subtropical forest share both core bacterial and fungal microbiome. Comparing our microbiome with the phyllosphere core bacterial microbiome detected in a neotropical forest [18] and in temperate forests [17] indicates that these three types of forests share a core bacterial microbiome with most dominant bacterial taxa. In addition, *Alphaproteobacteria* are the largest number of microbial groups in all these three types of forests. In our study, the phyllosphere fungal community is mainly composed of members of the phylum Ascomycota, which is similar to what has been found in tropical and temperate forests [19, 28, 74]. Additionally, the most common fungal classes observed across all plant families include *Dothidiomycetes*, *Sordariomycetes* and *Eurotiomycetes*, which is consistent with the finding in tropical forests [19]. Therefore, it is likely that most dominant taxa of core phyllosphere microbiomes are the same from tropical to temperate forests.

### Drivers of variation in phyllosphere microbial community composition and diversity

Many studies have reported that the plant taxa can drive the assemblage of phyllosphere microbiome in neotropical forests [18, 19, 38, 75] and temperate forests [17]. Our data indicate that in lower subtropical forests, host species identity is also the main driver of phyllosphere bacterial or fungal community structures. Moreover, for both the bacterial and fungal community structures, host species identity is more important than the factor season since season and its interaction with host species identity account for less variations than host species identity does. These suggest that evolutionary associations between host species and bacteria or fungi may play a key role in structuring phyllosphere microbial community so that although seasonality does have significant effects on microbial community assembly, the variation in community assembly between dry and wet seasons are smaller than that among host tree species. This is consistent with the findings in temperate forests where the variance explained by sampling time was small relative to the importance of host species identity [76]. As indicated by Laforest-Lapointe, Messier and Kembel [76], the reason may be that temporal changes during a growing season are not enough to overcome the influence of host species identity on community assembly once a community of bacteria successfully colonizes a leaf.

Our NMDS results indicate that there is a strong clustering of the phyllosphere microbial assemblage amongst trees by seasonality (Fig. 2a), thus the leaf microbial communities are temporally dynamic. However, the effect that different seasons have in driving bacterial community assembly is stronger in our subtropical forests than sampling times in the same growing season in temperate forests [17]. Although we do not know the exact reasons for such difference, one of them could be that the environmental differences between our contrasting sampling seasons in our study might be larger than those among sampling times within the same season in their study, and more different environments select more different bacteria. In the DHS plot, there are large differences in temperature, precipitation and light intensity between dry and wet seasons. The temperature in the wet season is much higher than that in the dry season, the precipitation in the wet season is four times that of the dry season [77], and the photosynthetically active radiation in the dry season is only about 70% of the wet season [78]. The apparent seasonal differentiation in phyllosphere microbial communities is consistent with the idea that environmental selective pressure on phyllospere communities due to abiotic conditions such as climate difference [76]. To test this hypothesis, further studies with sampling between and within seasons are necessary in both subtropical and temperate forests.

Kembel et al. [18] reported the evolutionary associations between host tree species and phyllosphere bacteria in tropical forests. We not only found such associations (Fig. 5a) but also the evolutionary associations between the hosts and phyllosphere fungi (Fig. 5b) in our lower subtropical forest. For example, in our study, *Alphaproteobacteria* are highly abundant on plants in Saxifragaceae, Rosaceae, and Leguminosae which belong to Rosales (Fig. 1a). However, *Acidobacteriia* have the highest abundance on plants in Pinaceae than all other families in our study (Fig. 1a). This may be because *Acidobacteriia* are oligotrophic bacteria and are generally dominant in harsh environments [79] where Pinaceae often grows. During the evolutionary process, *Acidobacteriia* have adapted to inhabiting tree canopies of Pinaceae. The adaptive between phyllosphere microbes and their host tree species needs further research [80].

Our results show that the phyllosphere bacterial community composition is driven by host traits, which is lower than that in neotropical forest [18]. In tropical forests, it is correlated with traits linked to plant resource uptake strategies [18, 72] and growth-mortality trade-off [81]. Similar correlations have been reported in temperate forests [17]. Our phyllosphere bacterial community structure is also linked to traits related to plant resource uptake strategy such as leaf N concentration and SLA (Fig. 3a) and defensive traits such as Phe concentration (Fig. 3b). Taken together, these results suggest that phyllosphere bacterial communities are shaped by the functional strategies of their plant hosts in that the factors driving the plant–microbe associations in the phyllosphere are similar across temperate, subtropical and tropical forests. Among them, the traits linked to plant resource uptake strategies are the most important traits that influence phyllosphere bacterial community structure in temperate, subtropical and tropical forests [17, 18].

We observed that phyllosphere fungal community structure is related to plant growth-mortality trade-off strategy (e.g., DBH, height, MR), and, like our bacterial communities, to plant resource uptake strategy (e.g., C, N, Ca) (Fig. 3c), as our bacterial communities. Whether the former relation also applies to other types of forests needs further studies.

Our data indicate that leaf Ca concentration is the most important factor among the traits and it is significantly negatively related to bacterial Shannon and Pielou indices (Fig. 4a, b). This may be attributed to that Ca is one of the components of cell walls that play an important role in blocking bacteria. Thus, host species with higher leaf Ca concentration would have lower phyllosphere bacterial Pielou and Shannon indices, which has been observed in tropical tree species [18]. We found that variation in fungal community composition was largely explained by host species identity while IV is the most powerful explanatory variable among the traits in explaining fungal Shannon and Pielou indices, but height and DBH are not significantly correlated to these indices. These findings indicate that, relative to bacteria, variations in community composition and diversity of phyllosphere fungi are more host species-specific.

## Conclusions

In this study, we demonstrate, for the first time, the natural subtropical tree phyllosphere bacterial and fungal communities across diverse tree species. Furthermore, we explored the roles of host species identity, host traits and seasonality on phyllosphere microbial community structure and diversity. Our key findings include: (1) phyllosphere of subtropical tree species share a “core microbiome”; (2) host species identity is a stronger driver of subtropical tree phyllosphere microbial communities than seasonality; (3) there is a strong clustering of the phyllosphere assemblage amongst trees by seasonality, and the seasonality effects are more on bacterial than fungal community assembly; and (4) there are significant evolutionary associations between host species and phyllosphere microbiome. Our findings suggest that subtropical tree phyllosphere bacterial and fungal communities vary with host species identity, traits and seasonality. The strong relationship between host plant importance in plant community and the phyllosphere fungal communities highlights the necessity of integrative studies towards incorporating host plant community assembly with phyllosphere microbial community assembly. Moreover, our results, together with the information on plant–microbe associations in the phyllosphere of tropical and temperate forests, can advance our understanding of the patterns and drivers of phyllosphere microbial community assembly in natural zonal forests at a global scale. It is also of interest to understand whether a phyllosphere core microbiome is shared by different types of forests and how they will vary under the scenarios of global change.

## Supplementary Information

**Additional file 1: Table S1.** Information for the 45 representative tree species in the Dinghushan 20-ha lower subtropical evergreen broad-leaved forest dynamics plot in Guangdong Province, China.

**Additional file 2: Table S2**. Abbreviations and units of plant traits measured and the references for their measurements.

**Additional file 3: Table S3.** Pearson correlation coefficients of the relationships between biodiversity metrics and plant traits.

**Additional file 4: Fig. S1.** Species accumulation curve (mean ± 95% confidence interval) of phyllosphere bacterial (**a**) and fungal (**b**) OTUs (97% sequence similarity cutoff) richness versus number of plant samples (2 per host species).

**Additional file 5: Fig. S2.** Venn diagram of phyllosphere bacterial (**a**) and fungal (**b**) OTUs in wet and dry seasons.

## Data Availability

All raw sequencing data have been submitted to the Genome Sequence Archive (GSA) database under the accession number CRA005533.

## Abbreviations

OUT: Operational taxonomic unit
PCR: Polymerase chain reaction
PERMANOVA: Permutational multivariate analysis of variance
RDA: Redundancy analysis
SLA: Specific leaf area
LDMC: Leaf dry matter content
A_mass_: Maximum CO_2_ assimilation rate per unit dry mass
WUE: Photosynthetic water use efficiency
C: Leaf carbon concentration
N: Leaf nitrogen concentration
P: Leaf phosphorus concentration
Ca: Leaf calcium concentration
K: Leaf potassium concentration
Si: Leaf silicon concentration
Phe: Total leaf phenolics concentration
Tan: Total leaf tannins concentration
Fla: Total leaf flavonoids concentration
DBH: Tree diameter at breast height
Height: Average tree height
GR: DBH relative growth rate from the year 2005 to the year 2015
MR: Tree mortality rate from the year 2005 to the year 2015
WD: Sapwood density
IV: Importance value
