# Supplementary material for: Phyllosphere bacterial and fungal communities vary with host species identity, plant traits and seasonality in a subtropical forest
Source: Environ Microbiome. 2022 Jun 9;17:29. doi: 10.1186/s40793-022-00423-3 (PMC9185928; doi:10.1186/s40793-022-00423-3)
Supplement: Supplementary file 2 — Additional file 2: Table S2. Abbreviations and units of plant traits measured and the references for their measurements. [file 40793_2022_423_MOESM2_ESM.docx]

Table S2 Abbreviations and units of plant traits measured and the references for their measurements

| Trait type | Trait | Abbreviation | Unit | Reference No. |
| --- | --- | --- | --- | --- |
| Leaf morphological traits | Specific leaf area | SLA | cm^2^ g^–1^ | (1) |
|  | Leaf dry matter content | LDMC | % | (1) |
| Leaf physiological traits | Maximum CO_2_ assimilation rate per unit dry mass | A_mass_ | nmol g^–1^ s^–1^ | (2) |
|  | Photosynthetic water use efficiency | WUE | mmol mol^–1^ | (2) |
| Leaf stoichiometric traits | Leaf carbon concentration | C | mg g^–1^ | (2) |
|  | Leaf nitrogen concentration | N | mg g^–1^ | (2) |
|  | Leaf phosphorus concentration | P | mg g^–1^ | (2) |
|  | Leaf calcium concentration | Ca | mg g^–1^ | (3) |
|  | Leaf potassium concentration | K | mg g^–1^ | (3) |
|  | Leaf silicon concentration | Si | mg g^–1^ | (4) |
| Leaf defense traits | Total leaf phenolics concentration | Phe | mg g^–1^ | (5) |
|  | Total leaf tannins concentration | Tan | mg g^–1^ | (6) |
|  | Total leaf ﬂavonoids concentration | Fla | mg g^–1^ | (7) |
| Stem traits | Tree diameter at breast height | DBH | cm | (8, 9) |
|  | Average tree height | Height | m | (8, 9) |
|  | DBH relative growth rate from the year 2005 to the year 2015 | GR | cm cm^–1^ year^–1^ | (10) |
|  | Tree mortality rate from the year 2005 to the year 2015 | MR | % | (10) |
|  | Sapwood density | WD | g cm^–3^ | (2) |
| Community-related trait | Importance value | IV | – | (8, 9) |

References

1. Pérezharguindeguy N, Díaz S, Garnier E, Lavorel S, Poorter H, Jaureguiberry P, et al. New handbook for standardised measurement of plant functional traits worldwide. Aust J Bot. 2013;61:167-234.

2. Zhu SD, Song JJ, Li RH, Ye Q. Plant hydraulics and photosynthesis of 34 woody species from different successional stages of subtropical forests. Plant Cell Environ. 2013;36:879-91.

3. Santos J, Oliva-Teles MT, Delerue-Matos C, Oliveira MBPP. Multi-elemental analysis of ready-to-eat "baby leaf" vegetables using microwave digestion and high-resolution continuum source atomic absorption spectrometry. Food Chem. 2014;151:311-6.

4. Proost J, Santoro R, Abu Jeriban S, Guiot I. Spectrophotometric determination of silicon in ultrapure, dilute hydrofluoric acid solutions. Microchem J. 2008;89:48-51.

5. Singleton V, Rossi JA. Colorimetry of total phenolics with phosphomolybdic-phosphotungstic acid reagents. Amer J Enol Viticult. 1964;16:144-58.

6. Porter LJ, Hrstich LN, Chan BG. The conversion of procyanidins and prodelphinidins to cyanidin and delphinidin. Phytochemistry. 1985;25:223-30.

7. Cao JG, Xia X, Chen XF, Xiao JB, Wang QX. Characterization of flavonoids from *Dryopteris erythrosora* and evaluation of their antioxidant, anticancer and acetylcholinesterase inhibition activities. Food Chem Toxicol. 2013;51:242-50.

8. Condit R. Tropical forest census plots : methods and results from Barro Colorado Island , Panama and a comparison with other plots. Berlin: Springer; 1998.

9. Ye WH, Cao HL, Huang ZL, Lian JY, Wang ZG, Li L, et al. Community structure of a 20 ha lower subtropical evergreen broadleaved forest plot in Dinghushan, China. J Plant Ecol (Chinese Ver). 2008;32:274-86. (in Chinese with English abstract).

10. Shen Y, Santiago LS, Shen H, Ma L, Lian JY, Cao HL, et al. Determinants of change in subtropical tree diameter growth with ontogenetic stage. Oecologia. 2014;175:1315-24.
